# Lack of Efficacy of Combined Antiangiogenic Therapies in Xenografted Human Melanoma

**DOI:** 10.1155/2012/794172

**Published:** 2011-10-05

**Authors:** Una Adamcic, Clorinda Castagna, Kanwal Minhas, Siranoush Shahrzad, Brenda L. Coomber

**Affiliations:** Department of Biomedical Sciences, Ontario Veterinary College, University of Guelph, Guelph, ON, N1G 2W1, Canada

## Abstract

Antiangiogenic therapy is theoretically a promising anticancer approach but does not always produce significant tumor control. Combinations of antiangiogenic therapies are therefore being investigated as strategies to enhance clinical benefit. Common targets for angiogenic blockade include endothelial specific receptors, such as Tie2/Tek, which signal blood vessel stabilization via recruitment and maturation of pericytes. Here, we report on the effects of targeted Tie2 antiangiogenic therapy (TekdeltaFc) in combination with nontargeted metronomic cyclophosphamide (LDM CTX) (reported to also act in antiangiogenic fashion) in xenografted human melanoma. Individually, these therapies showed transient antitumor activity, but, in combination, there was no significant reduction in tumor growth. In addition, while TekdeltaFc caused the expected increased pericyte coverage in treated blood vessels, LDM CTX alone or in combination with TekdeltaFc resulted in increased levels of VEGF production. Collectively, our data highlight the complexity of molecular interactions that may take place when antiangiogenic regimens are combined.

## 1. Introduction

Cytotoxic chemotherapeutic drugs may have antiangiogenic properties when administered metronomically at doses significantly lower than the maximum tolerated dose (MTD) and as such appear to have less severe or even absent cytotoxic side effects [1]. Cyclophosphamide (CTX), a nitrogen mustard alkylating agent, is clinically the most studied drug in a low-dose metronomic chemotherapy setting [2, 3]. In contrast to MTD, metronomic administration of low-dose cyclophosphamide induces selective apoptosis of genetically stable endothelial cells in tumors (hence circumventing drug resistance) [4, 5]. In addition, studies suggest that this effect is due to overproduction of Thrombospondin-1, a well-known, highly specific, and potent endogenous inhibitor of angiogenesis [6, 7]. Interestingly, an inverse relationship between Thrombospondin-1 and VEGF production has been reported in cultured ovarian epithelial cells and LDM-CTX has been shown to decrease VEGF levels in patients with breast cancer [8], suggesting a possible relationship between VEGF expression and LDM-CTX-induced Thrombospondin-1 expression. In general, the antitumor effects of cancers treated with LDM chemotherapy are even more pronounced when combined with an antiangiogenic inhibitor that targets endothelial cells specifically [9, 10]. Tie2 receptors are primarily found on endothelial cells and are constitutively expressed in normal vasculature [11]. Thus, it was surprising when our laboratory identified blood vessels within human cancers that lacked Tie2 expression. In particular, malignant melanoma had the highest percentage (~15%) of Tie2-negative blood vessels of all cancer types evaluated [12]. Differential expression of vascular growth factor receptors, such as Tie2, may be due to tumor microenvironment conditions such as hypoxia and hypoglycemia, which occur as a result of an imbalance between the oxygen supply and consumption and altered energy demand [13–15]. 

Heterogeneous expression of Tie2 in tumor vasculature suggested a role for Tie2 in tumor angiogenesis, however, elucidating the functional significance of Tie2 expression in tumors required the use of a specific Tie2 inhibitor TekdeltaFc, which is an artificial extracellular domain of Tie2 [16]. Since angiopoietins bind with high affinity to Tie2 extracellular domain, we reasoned that this inhibitor should efficiently interfere with angiopoietin-mediated Tie2 activity. In addition, we hypothesized that the tumor microenvironment is at least partially responsible for the lack of Tie2 expression observed in malignant melanoma blood vessels and investigated the possibility that Tie2 heterogeneity is due to severe hypoxia or hypoglycemia.

Excessive tumor angiogenesis has been associated with poor prognosis in metastatic melanoma [17]. Thus, antiangiogenic therapies targeting the tumor microvasculature have been intensively used in clinical trials in combination with the standard chemotherapeutic regimens. In one such study, antiangiogenic low-dose paclitaxel in combination with celecoxib caused significant disease stabilization of more than 6 months in 15% of metastatic melanoma patients [18]. Clinical benefits were also observed in melanoma patients treated with anti-VEGF therapy, bevacizumab (Avastin), combined with carboplatin and paclitaxel [19], or in combination with interferon-*α*-2b [20]. However, to date, the effect of Tie2 inhibition on melanoma angiogenesis, alone or in combination with other antiangiogenic strategies, has not yet been explored. 

Recent publications suggest a clear advantage of simultaneously using multiple antiangiogenic therapies in combination with metronomic, low-dose chemotherapy, thus targeting more than one endothelial cell signaling pathway [5, 21, 22]. To determine if the response to targeted antiangiogenic therapy such as Tie2 inhibition can be enhanced by nontargeted antiangiogenic therapy such as LDM CTX, we examined the impact of this combined approach on human malignant melanoma cancer cell xenografts. 

## 2. Methods

### 2.1. Cell Lines and Reagents

The human melanoma cell line WM239 was originally isolated from a patient's metastatic lesion [23]. Cells were maintained in Dulbecco's modified Eagle's Medium (Sigma-Aldrich) supplemented with 10% FBS (Invitrogen), sodium pyruvate (Invitrogen), and gentamicin (Invitrogen) in a humidified atmosphere at 37°C in 5% CO_2_. Tie2 inhibitor, murine TekdeltaFc, was provided by Amgen. Cyclophosphamide (CTX) was purchased from Sigma Aldrich.

### 2.2. Growth of Tumor Xenografts

All procedures described below were done according to the guidelines and recommendations of the Canadian Council of Animal Care and approved by the University of Guelph Local Animal Care Committee. Tumor xenografts were established in *RAG1^−^* immune-deficient mice [24] by injecting 100 *μ*L of 0.1% BSA/PBS solution containing 1 × 10^6^ WM239 melanoma cells subcutaneously into the right flank. Tumor growth was measured twice weekly and tumor size estimated using the equation: volume = length × width^2^  × 0.5. Once tumors reached at least 100 mm^3^, mice were randomly allocated into one of four treatment groups each containing 8 mice. Mice were treated for 14 days as follows: group 1 received 250 *μ*g TekdeltaFc as 250 *μ*L i.p. every 3 days; group 2 received low-dose cyclophosphamide in drinking water (equivalent to 30 mg/kg/day; water was changed twice weekly); group 3 received both TekdeltaFc every 3 days and low-dose cyclophosphamide in drinking water; group 4 control mice received 250 *μ*L i.p. injections of sterile PBS every 3 days and untreated drinking water. Tumor growth was measured for the duration of the trial every 3-4 days. 

One hour prior to euthanasia, mice were injected i.p. with 150 mg/kg Hypoxyprobe-1 (Chemicon International Inc.). Mice were euthanized by CO_2_ asphyxiation followed by cervical dislocation. Tumors were dissected from the surrounding tissue and cut into pieces, embedded in OCT cryomatrix (Fisher Scientific), and snap frozen in liquid nitrogen, fixed in 4% paraformaldehyde (USB Corporation) for 24 h and paraffin embedded, or snap frozen in liquid nitrogen and stored at −80°C for future protein isolation. 

### 2.3. Quantification of Tumor Hypoxic and Necrotic Areas

Paraformaldehyde-fixed paraffin-embedded 8 *μ*m thick sections were deparaffinized, and sodium citrate antigen retrieval (10 mM, pH = 6.0, boiled for 8 minutes then cooled in buffer at RT for 15 min) was performed. Following antigen retrieval, sections were washed and blocked first with Dako protein-free block (Dako) for 15 minutes and then with 5% normal goat serum (Sigma-Aldrich) for 30 minutes. Next, 3% hydrogen peroxide (Fisher Scientific) was used for 15 minutes to block endogenous peroxidase activity, then sections were washed and incubated in mouse Hypoxyprobe-1 antibody (Chemicon International) (1 : 50) overnight at 4°C, followed by goat antimouse biotinylated secondary antibody (1 : 200) for 30 minutes. Sections were washed and treated with R.T.U. Vectastain Elite ABC reagent (Vector) for 30 minutes followed by incubation with substrate reagent diaminobenzidine (DAB) for 2 minutes. Sections were then rinsed with water, counterstained using Mayer's hematoxylin solution (diluted 1 : 1 with water) (Sigma-Aldrich) for 1 minute, and mounted using Aquapolymount (Polyscience). In total, two blocks from each of twenty tumors were evaluated (five from each of the four treatment groups). Images were captured in a blinded fashion using 20x magnification objective of a Leica DMLB compound light microscope fitted with a Q imaging QICAM fast1394 digital camera using Q-Capture software, depending on the size of the tumor, certain sections were subdivided into one to four fields of view. Hypoxic regions were identified by strong brown reaction product, and necrotic regions were identified as those adjacent to hypoxic zones and lacking intact, well-defined nuclei (as seen with hematoxylin staining). Optimas 6.0 software (Optimas, Houston) was used to quantify areas of hypoxia and necrosis in each section and percentage of hypoxia/necrosis or hypoxia and necrosis per section area was calculated. 

### 2.4. Quantification of Tie2 Expression and Microvessel Density (MVD)

To evaluate Tie2 expression patterns as well as determine MVD, two tissue blocks from each tumor were assessed. Cryosections, 8 *μ*m thick, were cut using a cryostat adjusted to −20°C. Once cut, sections were stored at −80°C until further use. For immunofluorescent staining, sections were air dried at RT, fixed in cold methanol/acetone (50 : 50) for 10 min at −20°C, and then air dried. Then, sections were rehydrated in PBS and blocked using 10% normal goat serum (Sigma-Aldrich) for 1 h at room temperature followed by mouse anti-Tie2 (1 : 100; BD Biosciences) for 1 h at room temperature and incubation with goat antimouse Cy3 conjugated secondary antibody for 20 minutes (1 : 200; Jackson ImmunoResearch). Sections were blocked using Dako protein-free block (Dako) for 15 minutes, incubated overnight at 4°C using rat anti-CD31 antibody (1 : 50; Hycult Biotechnology), followed by donkey antirat FITC secondary antibody for 30 minutes (1 : 100; Jackson ImmunoResearch) and 2 minutes in DAPI (4′,6-diamidino-2-phenylindole) (Dako) nuclear stain. Slides were then washed briefly in water and mounted using Fluorescent Mounting media (Dako). Entire sections were examined by epifluorescence microscopy using the 20x objective in a semiblinded fashion. MVD was determined by dividing the total number of blood vessels per field of view, to obtain a value expressed as number of blood vessels per mm^2^.

### 2.5. Quantification of Blood Vessel Pericyte Coverage

To evaluate the degree of pericyte coverage of tumor blood vessels, two blocks from each tumor were assessed. Cryosections, 8 *μ*m thick, were fixed as previously described, then rehydrated in PBS, permeabilized using 0.05% Tween-PBS for 10 minutes and blocked using 10% normal goat serum (Sigma-Aldrich) for 30 minutes at RT. Sections were incubated in a mixture of rabbit anti-PDGFR-*β* (1 : 100; Cell Signaling Technology) and biotinylated rat anti-CD31 (1 : 60; Hycult) primary antibodies overnight at 4°C. Sections were washed in PBS and incubated in a mixture of goat antirabbit secondary antibody conjugated to Cy3 (1 : 200; Jackson ImmunoResearch) and streptavidin conjugated to Alexa350 (1 : 150; Invitrogen) for 40 minutes at room temperature followed by rabbit anti-desmin primary antibody for 30 minutes (1 : 100; Abcam, Cambridge, Mass, USA) conjugated to Alexa488 using the rabbit antibody labeling kit according to manufacturer's protocol (Invitrogen). Sections were mounted in Fluorescent Mounting Media (Dako) and examined using epifluorescence microscopy as previously described. Five random fields were captured, and blood vessels were enumerated as CD31^positive^/desmin^positive^/PDGFR-*β* ^positive^ or CD31^positive^/desmin^negative^/PDGFR-*β* ^negative^ and expressed as percentage per section.

### 2.6. Measurement of VEGF Levels in Tumor Xenograft Lysates

Commercially available human and mouse VEGF ELISA kits (both R & D Systems) were used to quantify VEGF levels in lysed tumor xenografts from each of the treatment groups (control, TekdeltaFc, LDM CTX and TekdeltaFc + LDM CTX). Briefly, frozen tumor pieces (3–5 per group) were defrosted on ice and lysed using a disposable tissue grinder and cell lysis buffer (Cell Signaling). Protein was collected as previously described and VEGF ELISA performed according to the manufacturer's protocol. Values were expressed relative to total tumor protein. 

### 2.7. Statistical Analysis

Calculation of preliminary summary statistics such as mean, standard deviation, and standard error was completed using Microsoft Excel (Microsoft). On all samples, Grubbs' test, also called the ESD method (extreme studentized deviate), was used to determine significant outliers. Once outliers, if any, were identified and omitted from the analysis; ANOVA was performed to determine the significance within and between groups (*P* < 0.05). Further, the least significant difference (Tukey) test was used if there was significant difference between groups. Data were presented as mean and standard error.

## 3. Results

### 3.1. Effects of LDM CTX and TekdeltaFc in Melanoma Xenografts

**Figure 1:**
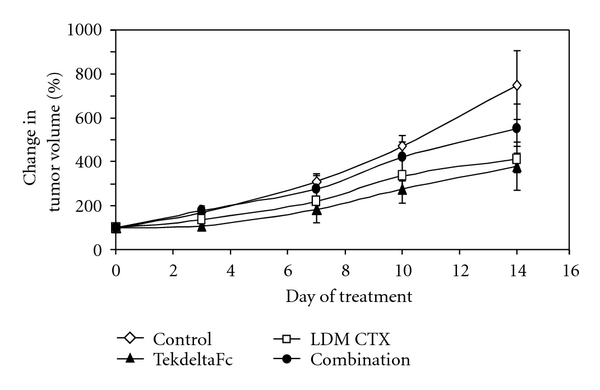
In vivo xenografts of human melanoma cancer cells treated with TekdeltaFc and LDM CTX. Relative tumor growth for each treatment group: Control, TekdeltaFc, low-dose metronomic cyclophosphamide (LDM CTX), and combination for two weeks. There were no statistically significant differences in tumor growth between treatment groups at any time points (*P* > 0.05).

**Figure 2:**
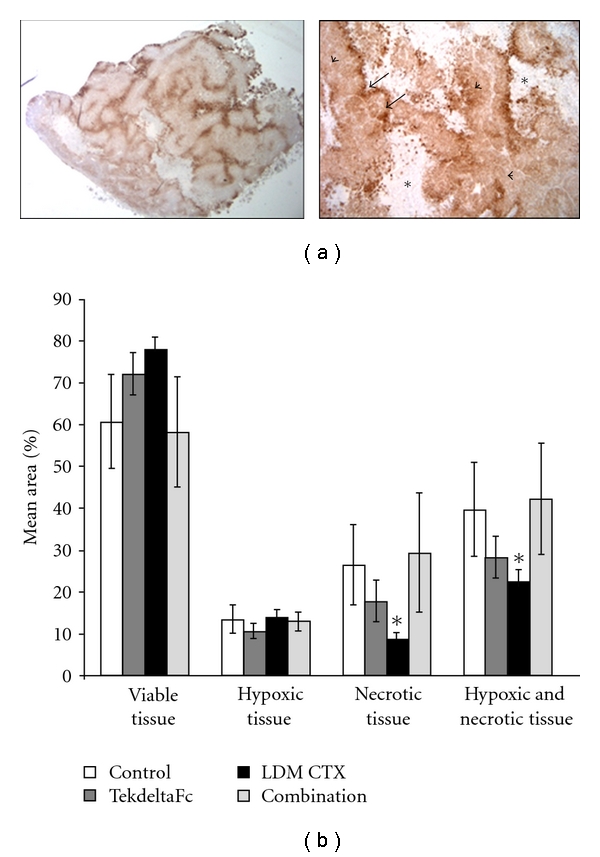
Quantification of hypoxic and necrotic areas in tumor xenografts treated with TekdeltaFc and LDM CTX. (a) Representative low (5x, left) and high (20x, right) magnification images of hypoxia immunostaining for Hypoxyprobe adducts and tissue necrosis in tumor sections. Dark brown areas are hypoxic (arrow), light brown areas are viable tumor (arrowhead), and areas lacking brown reaction product and containing degraded nuclei are necrotic tissue (asterisk). (b) Quantification of the mean percent viable tissue, hypoxia, necrosis, or hypoxia and necrosis in each treatment group, showing significant differences in proportions of necrotic, and necrotic plus hypoxic areas in LDM-CTX-treated tumors compared to control and combination (*P < 0.05).

While CTX LDM and TekdeltaFc showed a reduction in tumor growth compared to control, these differences were not statistically significant (Figure 1; *P* > 0.05). Surprisingly, there was also no additive effect when CTX LDM and TekdeltaFc were combined, as this dual target antiangiogenic regime also had no significant effect on tumor growth compared to control at any time point (Figure 1; *P* > 0.05). When tumor sections were evaluated for cellular responses (Figure 2(a)), we found no significant differences in the proportions of viable or hypoxic regions between treatment groups (Figure 2(b); *P* > 0.05). However, significantly lower amounts of tissue necrosis, and necrosis plus hypoxia were observed in tumors treated with CTX LMD when compared to control and combination-treated tumors (Figure 2(b); *P* < 0.05). 

**Figure 3:**
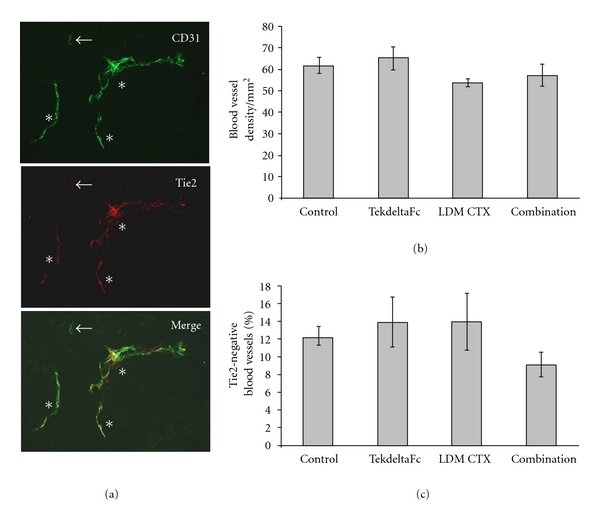
Dual immunofluorescence staining for CD31 and Tie2. (a) Tie2 was fluorescently labeled red using Cy3 while CD31 was fluorescently labeled green using FITC. Representative Tie2-positive blood vessels are marked with an asterisk while representative Tie2-negative blood vessels are labeled with arrows. (b) Quantification of average blood vessel densities (as number of CD31-positive vessels/mm^2^) between treatment groups. There were no statistically significant differences in blood vessel density between different treatment groups (*P* > 0.05). (c) Quantification of % Tie2-negative blood vessels in tumor xenografts treated with TekdeltaFc and/or LDM CTX. Graph depicts the average % of Tie2-negative blood vessels per treatment group; the difference between groups is not statistically significant (*P* > 0.05).

We quantified microvessel density (MVD) as well as the proportion of Tie2-negative vessels in tumor xenograft sections stained for CD31 and Tie2 (Figure 3(a)). The average number of Tie2 negative blood vessels was about 12.5%, which confirmed our previous finding of Tie2 vascular heterogeneity in melanoma [12]; there were no significant differences between treatment groups (*P* > 0.05). Although decreased MVD was observed in LDM-CTX-treated tumors compared to TekdeltaFc treatment group, this was not statistically significant (*P* > 0.05) (Figure 3(b)). 

### 3.2. TekdeltaFc Significantly Increased Pericyte Coverage of Tumor Blood Vessels Compared to Low-Dose CTX Treatment

**Figure 4:**
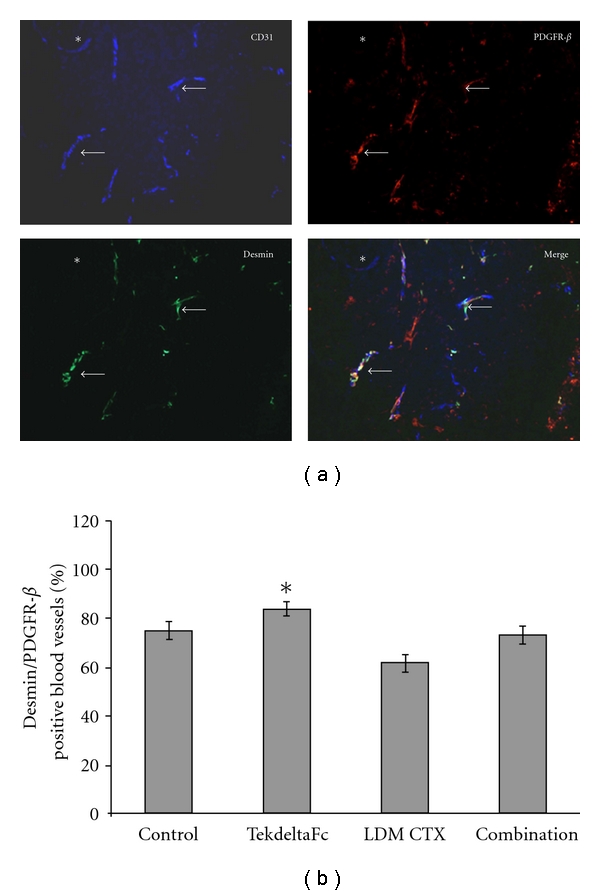
Immunostaining of blood vessels and pericytes in treated tumors. (a) Images show blood vessels immunofluorescently stained using antibodies to CD31 (Alexa 350; blue), desmin (Alexa 488; green), PDGFR-*β* (Cy3; red), and overlay of CD31/desmin/PDGFR-*β*. Asterisk marks blood vessel negative for mural cells (neither desmin nor PDGFR-*β*) while arrow indicates blood vessel with positive mural cell markers (desmin and PDGFR-*β* staining). (b) Quantification of blood vessel pericyte coverage in tumors treated with TekdeltaFc and/or LDM CTX. The percentage of “stable” blood vessels with pericyte coverage was significantly different between TekdeltaFc and all other treatment groups (*P < 0.05).

Triple immunofluorescence was utilized on frozen sections of xenografts with antibodies to desmin and PDGFR-*β* to detect vascular mural cells [25]. Vessels were categorized as Desmin/PDGFR-*β* ^positive^ or Desmin/PDGFR-*β* ^negative^ (Figure 4(a)). We observed a statistically significant difference (*P* < 0.05) between treatment groups. TekdeltaFc-treated tumors had statistically significant (*P* < 0.05) increased pericyte coverage compared to all other groups (Figure 4(b)). 

### 3.3. TekdeltaFc and LDM CTX Increased VEGF Expression

**Figure 5:**
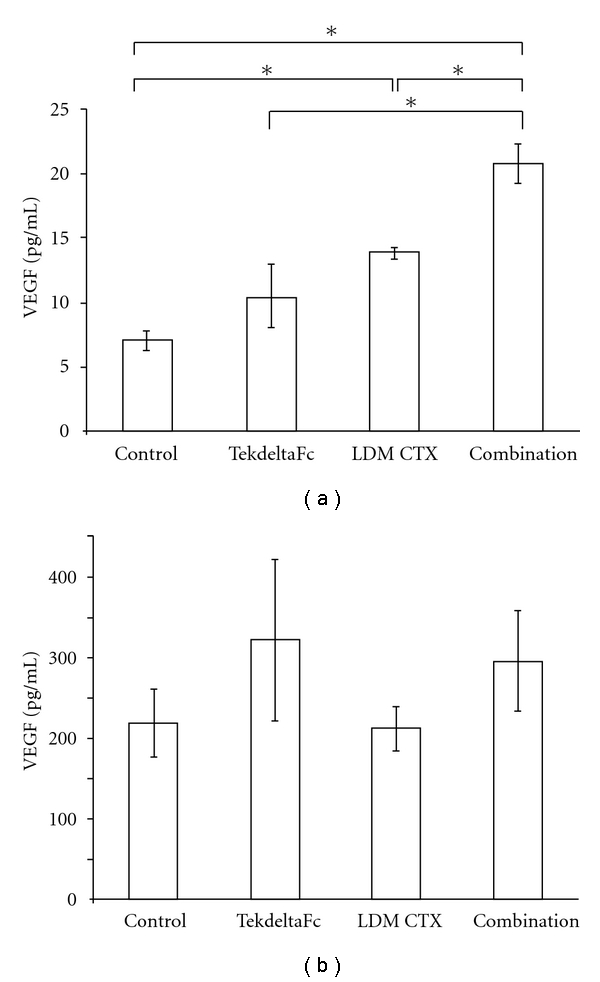
Expression of VEGF in tumor xenografts. Concentration of VEGF levels in tumor lysates measured by ELISA and expressed as pg/mL normalized to protein content in the tumor lysate. (a) There were significant differences in human VEGF levels between treatment groups (*P < 0.05). The lowest human VEGF concentration was detected in control tumors while the highest VEGF concentration was detected in combined treatment group, showing that TekdeltaFc and LDM CTX therapy both individually and in combination significantly upregulated melanoma cell VEGF expression. (b) There were no significant differences in murine VEGF between treatment groups (*P* > 0.05).

Tumor pieces from each treatment group were lysed and VEGF expression analyzed and expressed as pg/mL, normalized for total protein in the tumor lysate. Human VEGF levels were significantly increased (*P* < 0.05) in TekdeltaFc and LDM CTX combined treated tumors compared to all of the other treatment groups. In addition, human VEGF concentration was significantly higher (*P* < 0.05) in LDM CTX compared to control group (Figure 5(a)). Although murine VEGF levels were highest in LDM-CTX-treated tumors, ANOVA showed that overall there were no significant differences in murine VEGF levels (*P* > 0.05; Figure 5(b)).

## 4. Discussion

Tumor angiogenesis is an attractive therapeutic target, since it is shared by most commonly occurring, and perhaps all, types of human cancers [26]. Considering the importance of vascular growth in tumor progression, approaches targeting tumor endothelium using antiangiogenic therapies may provide a long-term and more effective control of disease compared to cytotoxic chemotherapy. One of the advantages of using antiangiogenic agents is that, under physiological conditions, normal endothelial cells are quiescent compared to tumor endothelial cells that are actively proliferating and migrating, which minimizes possible side effects on normal endothelium [27]. In addition, endothelial cells are genetically more stable than cancer cells and antiangiogenic agent delivery is less complicated by not having to penetrate large bulky masses. Finally, antiangiogenic therapies can have different modes of action—interfering with angiogenic ligands, their receptors or downstream signaling, upregulation/delivery of endogenous inhibitors, or by directly affecting tumor vasculature [28]. More recently, it has become apparent that cancer cells recruit a variety of bone-marrow-derived cells which are also able to contribute to the vasculature in direct and indirect ways [29].

 The mechanism of action of TekdeltaFc (the extracellular domain of murine Tie2/Tek receptor fused to the Fc portion of murine IgG) involves binding with high avidity to both Ang1 and Ang2 [16]. A similar inhibitory molecule, ExTek, was shown to function as a potent inhibitor of Tie2 by sequestering available angiopoietin, and by binding to Tie2 receptors, inhibiting phosphorylation and downstream signaling molecules related to cell survival [30–32]. Interestingly, ExTek decreased the number of lung metastasis in a murine melanoma model [33]. Other studies employing different versions of the Tie2 extracellular domain as an inhibitor achieved similar effects in different tumor models [34–36]. 

In our study, while LDM CTX had a more profound effect on tumor blood vessel density compared to TekdeltaFc treatment, these differences were not significant. LDM CTX may have had an indirect effect on tumor growth by significantly decreasing the mobilization of circulating endothelial cells from the bone marrow, as previously reported [37]. Interestingly, treatment of tumors with these antiangiogenic agents did not result in increased hypoxia/necrosis of the tumor tissue. This could be explained by some of our previous work which showed that WM239 cells can develop reduced vascular dependence and therefore enhanced survival even if their blood vessel density decreases [38] or by the fact that reduced vascular density actually represents a “normalization” of the vascular bed, accompanied by improved perfusion [39]. Such mechanisms are consistent with the fact that LDM-CTX-treated tumors had the lowest amount of necrotic tissue compared to control or combined treatment groups. 

We also observed that TekdeltaFc alone caused significant increases in desmin/PDGFR-*β* dual positive pericyte coverage of blood vessels compared to control, LDM CTX, or combination therapies. Antiangiogenic therapies have been previously shown to improve response to chemotherapy by inducing maturation of the blood vessels via increasing pericyte coverage [27, 40]. There is considerable variability in pericyte characteristics between tumor types, with some studies reporting that mature pericytes are PDGFR-*β* negative and desmin positive [40], while other studies report that tumor pericyte populations can have overlapping markers [41]. Interestingly, PDGFR-*β*-positive/desmin-negative cells were more likely to be detached from adjacent vessels [41]. In abnormal tumor vasculature, VEGF induces expression of Ang-2 from endothelial cells in the microvasculature [42, 43]. By binding to Tie2, Ang-2 becomes an autocrine regulator of endothelial cell function; whether it acts as an agonist [42, 44] or antagonist [45] is context dependent [46]. It is now known that in vitro Ang-2 inhibits the stabilizing effects of Ang-1, but weakly activates Tie2 if Ang-1 is absent [47]. In our tumor model, TekdeltaFc likely caused sequestering of abundantly expressed Ang-2, thus allowing Ang-1 to bind and phosphorylate Tie2, hence the observed increase in pericyte coverage. Vessel “normalization” has been associated with improved response to cytotoxic chemotherapy [21, 48], thus employing CTX at a maximally tolerated dose rather than a metronomic dose might be more effective.

Both TekdeltaFc and LDM CTX alone caused significant decreases in tumor volume at earlier time points, but they failed to do so after two weeks of treatment. This could be due to the fact that most advanced malignant tumors produce multiple angiogenic factors, and so targeting only angiopoietins (in TekdeltaFc treated tumors) or only VEGF (in LDM-CTX-treated tumors) may not be adequate for complete tumor control [8, 28, 49, 50]. Tumors may also have become resistant to TekdeltaFc or LDM CTX, allowing them to regrow after initial inhibition. Interestingly, tumor xenografts treated with TekdeltaFc and LDM CTX combination therapy grew at the same rate as controls, suggesting potential interference between these two therapies. This is in contrast to results with a neutralizing antibody against Ang2, which had potent antitumor effects in xenograft models, especially when combined with VEGF-targeted therapy [21]. Differences from our study may be due to the fact that, unlike anti-Ang2 antibody, TekdeltaFc affects Ang1 and Ang2 signaling. Differences may also be due to cancer type, as melanoma was not evaluated in the anti-Ang2 antibody study. Thus, although combination of antiangiogenic therapies is becoming a common practice [2, 22, 50, 51], our studies provide evidence that interactions may be complex and tumor type dependent. 

Surprisingly, treated tumors in our study contained significantly higher levels of human VEGF than control tumors, and combined treated tumors contained the highest amount, consistent with a proangiogenic environment observed at the end of this trial. One of the modes of resistance to antiangiogenic therapy, usually occurring after a transient response phase, is upregulation of alternative proangiogenic pathways in tumors, such as fibroblast growth factor 1 and 2, ephrin A1, or Ang 1. These presumably compensate for the inhibited pathway and allow tumor regrowth [52]. In one such study, patients treated with VEGFR inhibitor cediranib had an increase in FGF2 expression in their relapse phase after successful but transient response [53]. It has also been shown in clinical trials that tyrosine kinase inhibitors can transiently increase the levels of proangiogenic factors, such as VEGF [54], as we observed in our present study. In fact, increases in proangiogenic factor VEGF in the presence of antiangiogenic Tie2 inhibitor probably account for the lack of an observed decrease in blood vessel density in our treated tumors. Collectively, these data support the idea that two different antiangiogenic therapies may interact to stabilize the microvasculature, thus preventing vessel regression and tumor inhibition, an outcome that suggests that caution should be taken in designing such antiangiogenic combinations.
